# PI4K2A deficiency causes innate error in intracellular trafficking with developmental and epileptic‐dyskinetic encephalopathy

**DOI:** 10.1002/acn3.51634

**Published:** 2022-07-25

**Authors:** Hormos Salimi Dafsari, Joshua G. Pemberton, Elizabeth A. Ferrer, Tony Yammine, Chantal Farra, Mohammad Hasan Mohammadi, Ehsan Ghayoor Karimiani, Narges Hashemi, Mirna Souaid, Sandra Sabbagh, Paria Najarzadeh Torbati, Suliman Khan, Emmanuel Roze, Andres Moreno‐De‐Luca, Aida M. Bertoli‐Avella, Henry Houlden, Tamas Balla, Reza Maroofian

**Affiliations:** ^1^ Department of Pediatrics Faculty of Medicine and University Hospital Cologne, University of Cologne Kerpener Str. 62 50937 Köln Germany; ^2^ Max‐Planck‐Institute for Biology of Ageing and CECAD Cologne Germany; ^3^ Evelina Children's Hospital, Guy's & St Thomas' NHS Foundation Trust London UK; ^4^ Section on Molecular Signal Transduction, Program for Developmental Neuroscience, Eunice Kennedy Shriver NICHD National Institutes of Health Bethesda Maryland USA; ^5^ Medical Genetics Unit Saint Joseph University Beirut Lebanon; ^6^ Department of Genetics Hotel Dieu de France Medical Center Beirut Lebanon; ^7^ Department of Pediatrics Zabol University of Medical Sciences Zabol Iran; ^8^ Molecular and Clinical Sciences Institute St. George's, University of London Cranmer Terrace London UK; ^9^ Department of Medical Genetics Next Generation Genetic Polyclinic Mashhad Iran; ^10^ Department of Pediatric Neurology, Faculty of Medicine Mashhad University of Medical Sciences Mashhad Iran; ^11^ CENTOGENE GmbH Rostock Germany; ^12^ CNRS, INSERM, Institut du Cerveau (ICM) Sorbonne Université Paris 75013 France; ^13^ DMU Neurosciences Hôpital de la Pitié‐Salpêtrière, Assistance Publique‐Hôpitaux de Paris Paris 75013 France; ^14^ Department of Radiology, Diagnostic Medicine Institute Autism & Developmental Medicine Institute, Genomic Medicine Institute, Geisinger Danville Pennsylvania USA; ^15^ Department of Neuromuscular Diseases UCL Queen Square Institute of Neurology Queen Square London UK

## Abstract

**Objective:**

Intracellular signaling networks rely on proper membrane organization to control an array of cellular processes such as metabolism, proliferation, apoptosis, and macroautophagy in eukaryotic cells and organisms. Phosphatidylinositol 4‐phosphate (PI4P) emerged as an essential regulatory lipid within organelle membranes that defines their lipid composition and signaling properties. PI4P is generated by four distinct phosphatidylinositol 4‐kinases (PI4K) in mammalian cells: PI4KA, PI4KB, PI4K2A, PI4K2B. Animal models and human genetic studies suggest vital roles of PI4K enzymes in development and function of various organs, including the nervous system. Bi‐allelic variants in *PI4KA* were recently associated with neurodevelopmental disorders (NDD), brain malformations, leukodystrophy, primary immunodeficiency, and inflammatory bowel disease. Here, we describe patients from two unrelated consanguineous families with PI4K2A deficiency and functionally explored the pathogenic mechanism.

**Methods:**

Two patients with PI4K2A deficiency were identified by exome sequencing, presenting with developmental and epileptic‐dyskinetic encephalopathy. Neuroimaging showed corpus callosum dysgenesis, diffuse white matter volume loss, and hypoplastic vermis. In addition to NDD, we observed recurrent infections and death at toddler age. We further explored identified variants with cellular assays.

**Results:**

This clinical presentation overlaps with what was previously reported in two affected siblings with homozygous nonsense *PI4K2A* variant. Cellular studies analyzing these human variants confirmed their deleterious effect on PI4K2A activity and, together with the central role of PI4K2A in Rab7‐associated vesicular trafficking, establish a link between late endosome‐lysosome defects and NDD.

**Interpretation:**

Our study establishes the genotype–phenotype spectrum of PI4K‐associated NDD and highlights several commonalities with other innate errors of intracellular trafficking.

## Introduction

Eukaryotic cells compartmentalize their cellular functions using organelles that are defined by membranes with unique lipid and protein composition as well as signaling properties. Transport of proteins, lipids and other soluble molecules either through vesicular or other intracellular transport pathways ensure proper dynamic adaptation of cells to changing environments. Inositol phospholipids (PPIns) represent a small fraction of cellular phospholipids with important roles in the control of cellular processes.^1^

While phosphatidylinositol 4‐phosphate (PI4P) has long been viewed solely as an immediate precursor of the regulatory lipid, PI(4,5)P_2_, studies in the last 15 years have clearly demonstrated that PI4P is a regulatory lipid on its own right, which functions specifically in the Golgi apparatus and endosomal membranes where it controls vesicular trafficking.^1^ PI4P gradients established at membrane contact sites between opposing organelle membranes have also recently emerged as a driving force to support non‐vesicular transport of structural lipids against concentration gradients.^2^ This paradigm‐shifting discovery has placed PI4P in the center of the lipid homeostatic cellular machinery. Mammalian PI4P is generated by four distinct phosphatidylinositol 4‐kinases (PI4Ks): PI4KA, PI4KB, PI4K2A, PI4K2B. While PI4KA establishes the PI4P gradient between plasma membrane (PM) and endoplasmic reticulum (ER), other PI4K enzymes generate PI4P gradients between Golgi‐ER or endosome‐ER interfaces, hence controlling lipid transport within these membrane compartments. Murine studies showed severe myelination defects in knockout of either PI4KA or PI4KB in Schwann cells^3^, ^4^ and spinocerebellar degeneration with lack of *Pi4k2a*.^5^ In light of these findings, the common thread between PI4K enzymes is their critical involvement in establishing the lipid landscape of mammalian cells, especially within neuronal development and plasticity.

The wider availability of massively parallel sequencing has aided the identification of such disease‐associated genes from intracellular trafficking pathways. Recent studies described 22 affected individuals with bi‐allelic variants in the phosphatidylinositol 4‐kinase alpha gene (*PI4KA*, OMIM *600286) causing a spectrum of neurodevelopmental disorder (NDD), brain malformations, contractures, hypomyelinating leukodystrophy, inflammatory bowel disease (IBD), and primary immunodeficiency (PID).^6^, ^7^, ^8^

To this date, only two homozygous variants in the phosphatidylinositol 4‐kinase type 2 alpha gene (*PI4K2A*) were reported to be associated with NDD phenotypes.^9^, ^10^ The first and most striking report showed homozygous nonsense variants in *PI4K2A* in two affected siblings presenting with NDD, epilepsy, myoclonus, and akathisia. In a subsequent study, a homozygous missense variant of uncertain significance was suggested to be responsible for some features in a case with NDD and metabolic cutis laxa. In the present study, we describe two patients from unrelated consanguineous families with PI4K2A deficiency characterized by developmental encephalopathy with hyperkinetic movement disorders, and epilepsy. We further examine whether the pathogenic variants found in these two index cases have an effect on intracellular vesicular trafficking.

## Materials and Methods

### Clinical and genomic investigations

**Figure 1:**
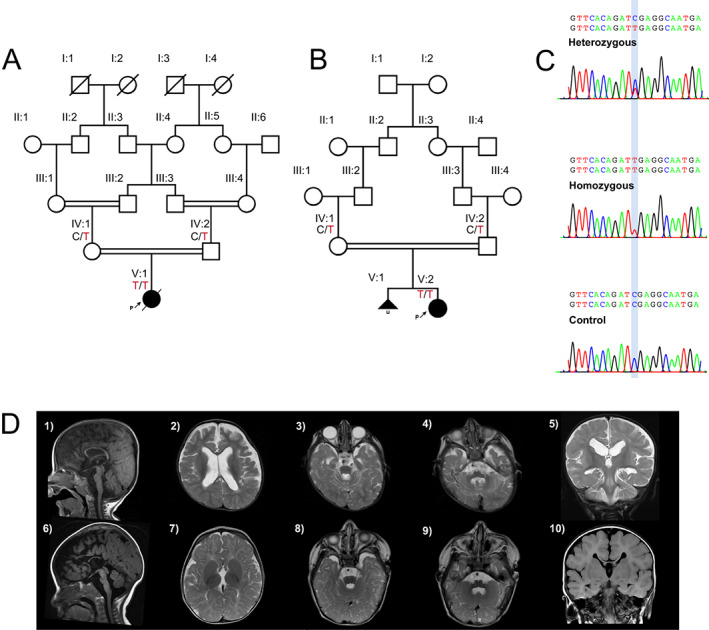
Molecular, clinical and neuroimaging findings in patients with bi‐allelic variants in the phosphatidylinositol 4‐kinase type 2 alpha gene (*PI4K2A*). (A) Pedigree of family 1 showing consanguinity and genotypes of tested individuals. (B) Pedigree of family 2 showing consanguinity and genotypes of tested individuals. (C) Examples of heterozygous, homozygous and control/wildtype forms of the genetic locus in *PI4K2A*. Created with PolyPeakParser. (D) Neuroimaging findings of individuals with *PI4K2A* pathogenic variants. Brain MRI findings of cases 1 (D1–5) and 2 (D6–10). Sagittal T1‐weighted images (D1, D6) showed dysgenesis of corpus callosum in case 1 (D1) and dysgenesis of corpus callosum with markedly hypoplastic and foreshortened body and underdeveloped rostrum, genu, and splenium with secondary radial configuration of the paramedian gyri with absent cingulate gyri in case 2 (D6). Both cases had hypoplastic anterior commissure and massa intermedia, hypoplastic brainstem predominantly involving the pons, hypoplastic vermis with widening of the foramen of Magendie, and mega cisterna magna (D1, D6). Axial T2‐weighted images (D2, D3, D4, D7, D8, and D9) and coronal T2‐ (D5) and T1‐ (D10) weighted images revealed white matter volume loss (D2, D6, D7, D10), hypoplastic superior cerebellar peduncles (D3, D8), and hypoplastic middle cerebellar peduncles (D4, D9). Case 1 had ventriculomegaly and widened anterior interhemispheric fissure (D2). Case 2 had agenesis of the septum pellucidum and asymmetrically rotated thalami (D7). See Table 1 for description of additional neuroimaging findings. [Colour figure can be viewed at wileyonlinelibrary.com]

In this study, we evaluated two independent consanguineous families of Iranian and Lebanese origin (Fig. 1A and B). The study was approved by the institutional ethics committees of the participating centers and written informed consent was obtained from the families in accordance with the Declaration of Helsinki. Detailed clinical features, family history, EEG recordings and brain magnetic resonance imaging (MRI) studies were carefully reviewed by a group of clinical geneticists, pediatric neurologists, and a neuroradiologist. Clinical and research exome sequencing and dideoxy sequencing segregation analysis were performed in each family according to previously published studies.^11^ The patient from family 1 was sequenced with proband‐only whole exome sequencing (WES). The patient from family 2 was sequenced by trio WES (patient, mother, father).

### Reagents

Stock solutions of all reagents were dissolved in the indicated solvent and stored in small aliquots at −20°C. GSK‐A1, a PI4KA‐selective inhibitor,^12^ was prepared at 100 μmol/L in DMSO, while Coelenterazine h (Regis Technologies, Morton Grove, IL, USA) was dissolved in 100% ethanol (vol/vol) at 5 mmol/L.

### Cell culture

HEK293‐AT1 cells, which stably express the rat AT_1a_ angiotensin II receptor,^13^ or PI4K2A knockout (K/O) cells (Clone #26; HEK293‐AT1 background^14^) were cultured in Dulbecco's Modified Eagle Medium (DMEM‐high glucose; Gibco, Life Technologies, Carlsbad, CA, USA) containing 10% (vol/vol) FBS and supplemented with a 1% solution of penicillin/streptomycin (Gibco, Life Technologies). Cell lines were maintained at 37°C and 5% CO_2_ in a humidified atmosphere and regularly tested for *Mycoplasma* contamination using a commercially available detection kit (InvivoGen). Additionally, after thawing, cell cultures were treated with plasmocin (InvivoGen, San Diego, CA, USA) at 500 μg/mL for the initial three passages (6–9 days) and supplemented with 5 μg/mL of the prophylactic for all subsequent passages.

### DNA constructs

Plasmids were constructed by standard restriction cloning using enzymes from New England Biolabs, while site‐directed mutagenesis was done using the QuikChange II kit (Agilent, Santa Clara, CA, USA). Truncations and point variants were verified using standard Sanger sequencing (Psomagen, Rockvilla, MD, USA). EGFP‐PI4K2A and the truncated protein EGFP‐PI4K2A‐R309^STOP^ were generated by PCR amplification (PfuUltra II Hotstart; Agilent) from PI4K2A‐HA^15^ and inserted into the pEGFP‐C1 vector (Clontech, TaKaRa, Kyoto, Japan) using XhoI and HindIII restriction sites. For the PCR reaction, a shared forward primer was used (5′‐CTCGAGCTATGGACGAGACGAGCCCACTAGTG‐3′) together with unique reverse primers to generate either the full‐length (Residues 1–479; 5′‐AAGCTTTTACCACCATGAAAAGAAGGGCTTCC‐3′) or truncated (Residues 1–309; 5′‐AAGCTTTTAATCAGTGTTGCGGATGATGTAATCCAG‐3′) PI4K2A inserts. EGFP‐PI4K2A was subsequently used as the template for site‐directed mutagenesis and the generation of the EGFP‐PI4K2A‐Arg275Trp protein (Forward Primer: 5′‐CTATTGGCTGTGGCGTTTTGAAGC‐3′; Reverse Primer: 5′‐AAAACGCCACAGCCAATAGTCTGC‐3′). The levels of PI4P in Rab7‐positive membrane compartments were measured using the bioluminescence resonance energy transfer (BRET)‐based sLuc‐P4M2x‐T2A‐mVenus‐Rab7 biosensor described previously.^14^ Additionally, to prevent any interference with the BRET signal, EGFP was replaced with the iRFP coding sequence from piRFP‐C1 in EGFP‐PI4K2A, EGFP‐PI4K2A‐Arg309Ter, and EGFP‐PI4K2A‐Arg275Trp using AgeI and NotI restriction sites.

### Live‐cell confocal microscopy

HEK293‐AT1 cells (3 × 10^5^ cells/dish) were plated with a final volume of 1.5 mL on 29 mm circular glass‐bottom culture dishes (#1.5; Cellvis, Mountain View, CA, USA) pre‐coated with 0.01% poly‐L‐lysine solution (Sigma, St. Louis, MO, USA). Plated cells were allowed to attach overnight prior to transfection with plasmid DNAs (0.1–0.2 μg/well) using Lipofectamine 2000 (2–5 μL/well; Invitrogen, Waltham, MA, USA) within a small volume of Opti‐MEM (200 μL; Invitrogen). Lipofectamine‐complexed DNA was incubated together with the cells for 4–6 h before being exchanged for complete culture medium. After 18–20 h of transfection, cells were incubated in 1 mL of modified Krebs‐Ringer solution (containing 120 mmol/L NaCl, 4.7 mmol/L KCl, 2 mmol/L CaCl_2_, 0.7 mmol/L MgSO_4_, 10 mmol/L glucose, 10 mmol/L HEPES, and adjusted to pH 7.4) and images were acquired at room temperature using a Zeiss LSM 880 (63x/1.40 N.A. Plan‐Apochromat Oil DIC M27 Objective) laser‐scanning confocal microscope together with the ZEN software system (Carl Zeiss Microscopy, Jena, Germany).

### BRET measurements

BRET‐based measurements of PI4P levels within Rab7‐positive membrane compartments of intact cells has been described in detail previously.^14^ Briefly, measurements were made at 37°C using a Tristar2 LB 942 Multimode Microplate Reader (Berthold Technologies, Bad Wildbad, Germany) with customized emission filters (540/40 and 475/20 nm). PI4K2A K/O cells (0.75 × 10^5^ cells/well) were seeded in a 200 μL total volume to white‐bottom 96 well plates pre‐coated with 0.01% poly‐l‐lysine solution (Sigma) and cultured overnight. Cells were then transfected with 0.25 μg of the sLuc‐P4M2x‐T2A‐mVenus‐Rab7 BRET biosensor and 0.01 μg of either iRFP‐PI4K2A, iRFP‐PI4K2A‐Arg309Ter, iRFP‐PI4K2A‐Arg275Trp, or the piRFP‐C1 empty vector using Lipofectamine 2000 (1 μL/well) within OPTI‐MEM (40 μL). Lipofectamine‐complexed DNA was incubated together with the cells for 4–6 h before being exchanged for complete culture medium. At between 20–24 h post‐transfection, cells were washed once before being incubated for 30 min in 50 μL of modified Krebs‐Ringer buffer (containing 120 mmol/L NaCl, 4.7 mmol/L KCl, 2 mmol/L CaCl_2_, 0.7 mmol/L MgSO_4_, 10 mmol/L glucose, 10 mmol/L HEPES, and adjusted to pH 7.4) at 37°C in a CO_2_‐independent incubator. After the pre‐incubation period, the cell‐permeable luciferase substrate, coelenterazine h (40 μL, final concentration 5 μmol/L), was added and a 5 min baseline BRET measurement (30 sec/cycle) was recorded. The plates were then quickly unloaded for manual addition of GSK‐A1 (30 nmol/L) or a DMSO vehicle control, which were prepared in a 10 μL volume of the modified Krebs‐Ringer solution. Measurements were carried out in triplicate wells and repeated as three independent experiments. From each well, the BRET ratio was calculated by dividing the 530 and 485 nm intensities, which were then normalized to the baseline measurement. To facilitate the pooling of data from individual wells and between replicate experiments, the raw BRET ratios were processed by using a simple moving average with a four‐cycle interval across the kinetic. For each iRFP‐PI4K2A variant or the iRFP‐C1 control, the processed BRET ratios obtained from GSK‐A1‐treated wells were normalized to an internal DMSO vehicle control.

## Results

### Clinical and neuroimaging findings

Table 1 shows the clinical and neuroimaging findings in our two cases. Patient 1 was a female patient born to 2nd degree consanguineous Iranian parents who was presented to our specialty clinic with severe neurodevelopmental delay, irritability, movement disorders, epilepsy, and failure to thrive at 18 months of age. The patient was born at full term (38 weeks) with a weight at 2200 g (1st percentile) and head circumference at 34 cm (44th percentile) without relevant abnormalities in perinatal adaptation. She never attained considerable milestones in motor, intellectual or speech development. At the age of 16 months, she first showed intractable myoclonic and tonic seizures. Treatment with clobazam for 1.5 years had moderate response while there were only intermittent improvements on treatment with levetiracetam, primidone, vigabatrin, topiramate, and phenobarbital. Neurological examination showed distinct muscular hypotonia and atrophy, intermittent dystonic postures of the upper limbs with clenched fist and thumb adduction, spasticity of limbs and orofacial dyskinesia (see Video S1). EEG showed slow background, bursts of central theta frequency waves, and frontotemporal spike–wave‐bursts. Brain MRI showed marked dysgenesis of corpus callosum. We also observed hypoplastic anterior commissure and massa intermedia, diffuse white matter volume loss with ventriculomegaly, widened anterior interhemispheric fissure, slightly rotated thalami, and hypoplastic brainstem predominantly involving the pons, hypoplastic vermis with widening of the foramen of Magendie, hypoplastic superior and middle cerebellar peduncles, and a mega cisterna magna. Standard metabolic tests (MS/MS and organic acids in urine) and karyotype were normal. The patient showed recurrent severe bouts of infection with 16 hospitalizations within 3 years, failure to thrive (height, weight, and head circumference all below 1st percentile at 34 months), and recurrent constipation which might point towards PID. The patient died at 3 years and 10 months of age.

**Table 1:** Epidemiological, neurological, musculoskeletal, neuroimaging, and other clinical phenotypes in two families with bi‐allelic variants in the phosphatidylinositol 4‐kinase type 2 alpha gene (*PI4K2A*).

Categories	Alkhater et al., patient II:8	Alkhater et al., patient II:9	Family 1	Family 2
Genomic variant	NM_018425.3:c.65C>A; p.(Ser22Ter)	NM_018425.3:c.65C>A; p.(Ser22Ter)	NM_018425.3:c.925C>T p.(Arg309Ter)	NM_018425.3:c.925C>T p.(Arg309Ter)
Zygosity	Homozygous	Homozygous	Homozygous	Homozygous
Allele frequency in human population adatabases including *UKBB, gnomAD(v2.1.1& v3.1.2), QSG, ESP, Iranome, GME Variome, Geno2MP* and Centogene	Absent	Absent	Absent	Absent
Epidemiological data				
Sex	Male	Male	Female	Female
Consanguinity of parents	Yes (first degree cousins)	Yes (first degree cousins)	Yes (first degree cousins)	Yes
Ethnic background	Saudi Arabian	Saudi Arabian	Iranian	Lebanese
Age (years) at last follow up	14 years	9 years	2 years and 10 months, died at 3 years and 10 months	2 years
Family history	No	No	No	A case of miscarriage (CGH revealed 45,X0)
Prenatal and perinatal history	No	No	No	Hypoplastic corpus callosum detected in utero
Anthropometric data				
Height at last follow‐up (cm)	N/A	N/A	74 (<1 perc., z=‐5.47)	80 (1. perc., z=‐2.32)
Weight at last follow‐up (kg)	N/A	N/A	5 (<1 perc., z=‐8.66)	10 (4. perc., z=‐1.72)
Head circumference at last follow‐up (cm)	N/A	N/A	42 (<1 perc., z=‐6.87)	48 (10 perc., z=‐1.26)
Gestational age at birth (weeks)	N/A	N/A	38	38
Length at birth (cm)	N/A	N/A	N/A	48 (9. perc., z=‐1.35)
Weight at birth (g)	N/A	N/A	2200 (1. perc., z=‐2.55)	3325 (51. perc., z=0.03)
Head circumference at birth (cm)	N/A	N/A	34 (26. perc., z=‐0.64)	35 (53. perc., z=0.07)
Failure to thrive	Yes	Yes	Yes	Yes
Development				
Motor delay	Yes	Yes	Yes	Yes
Hypotonia in infancy	No	No	Yes	Yes
Unsupport sitting	Yes	Yes	Never attained	Never attained
Walking	Never attained	Never attained	Never attained	Never attained
Speech delay	Yes	Yes	Yes	Yes
First mono‐syllabic words	Never attained	Never attained	Never attained	Never attained
Number of words	None	None	None	None
Nonverbal communication	N/A	N/A	Fixation and following with eyes	Fixation and following with eyes
Degree of intellectual disability	N/A	N/A	Profound	N/A
Peforms activities of daily living	No	No	No	N/A
Regression	Yes	Yes	No	N/A
Behavioral characteristics				
ADHD	No	No	No	No
Autism	Unknown	Unknown	Unknown	Unknown
Irritability, and akathisia	Yes	Yes	Yes	Yes
Disturbed sleep	Yes	Yes	Yes	Yes
Aggressive/self‐harm	No	No	No	No
Other behavioral and psychiatric symptoms	None	None	None	None
Seizures				
Seizure type	Generalized	Generalized	Tonic, myoclonic (30 sec each, intractable)	Epileptic spasms
Age of onset	9 years	9 years	16 months	6 months
Clustering	No	No	No	No
EEG	Slow background, bursts of central theta frequency waves, no frank epileptiform discharges	Slow background, bursts of central theta frequency waves, no frank epileptiform discharges	Slow background, bursts of central theta frequency waves, frontotemporal spike–wave‐bursts	Hypsarrhythmia, diffuse delta frequency
AEDs	Levetiracetam (good response)	Levetiracetam (good response)	Mediocre response with Clobazam, for 1.5 years. Only initial good response with Levetiracetam for 6 m, Primidone for 4 m, Vigabatrin for 11 m, Phenobarbital and Topiramate	Vigabatrin, Topiramate, Lamotrigine all with mediocre till no response
Neurological examination				
Deep tendon reflexes	Brisk	Brisk	3+ (increased)	3+ (increased)
Muscle weakness	Axial hypotonia	Axial hypotonia	Axial hypotonia	Axial hypotonia
Muscle atrophy	No	No	Yes	Yes
Spasticity	Unknown	Unknown	Yes	Yes
Ataxia	No	No	No	No
Choreoathetosis	No	No	No	No
Myoclonus	Yes	Yes	No	No
Dystonia	Episodic arm dystonia (improved with Clonazepam)	Episodic arm dystonia (improved with Clonazepam)	Episodic arm dystonia	No
Tremor	No	No	No	No
Limb contractures	Pes cavus	Pes cavus	Bilateral thumb contractures	Bilateral thumb contractures
Dyskinetic movements	Orofacial dyskinesia	Orofacial dyskinesia	Orofacial dyskinesia	Orofacial dyskinesia
Peripheral neuropathy	Yes	Yes	Unknown	Unknown
Gait abnormalities	Non ambulatory	Non ambulatory	Non ambulatory	Non ambulatory
Joint hypermobility	No	No	No	No
Abnormal spine curvatures	No	No	No	No
Other neurological findings	Opisthotonic posturing (improved with Clonazepam)	Opisthotonic posturing (improved with Clonazepam)	None	None
Investigations				
Metabolic	Cerebrospinal fluid neurotransmitter profile normal	Cerebrospinal fluid neurotransmitter profile normal	MS/MS from blood spots normal, organic acids in urine normal	MS/MS from blood spots normal, organic acids in urine normal
Genetic	None	None	Karyotype normal	None
Neuroimaging				
Brain MRI abnormalities	Hypoplasia of corpus callosum, loss of gyral infolding of cingulate gyri, unusual thickened appearance to lamina terminalis, small pons and brainstem, small mega cisterna magna	N/A	Dysgenesis of corpus callosum. Hypoplastic anterior commissure and massa intermedia. Diffuse white matter volume loss. Ventriculomegaly. Widened anterior interhemispheric fissure. Slightly rotated thalami. Hypoplastic brainstem predominantly involving the pons. Hypoplastic vermis with widening of the foramen of Magendie. Hypoplastic superior and middle cerebellar peduncles. Mega cisterna magna	Dysgenesis of the corpus callosum with markedly hypoplastic and foreshortened body and underdeveloped rostrum, genu, and splenium. Secondary radial configuration of the paramedian gyri with absent cingulate gyri. Widened anterior interhemispheric fissure. Hypoplastic anterior commissure and massa intermedia. Agenesis of the septum pellucidum. Asymmetrically rotated thalami. Diffuse white matter volume loss. Hypoplastic brainstem predominantly involving the pons. Hypoplastic vermis with widening of the foramen of Magendie. Hypoplastic superior and middle cerebellar peduncles. Mega cisterna magna
Miscellaneous				
Structural congenital abnormalities	Hypotelorism, small eyes, micrognathia, prominent ears	Hypotelorism, small eyes, micrognathia, prominent ears	High arched palate	No
Ophthalmologic abnormalities	No	No	No	No
Hearing impairment	No	No	ABR: normal	No
Recurrent infections	No	No	Yes (16 severe infections with hospitalizations), but no specific immune diagnostics before death	No
Cardiovascular abnormalities	No	No	No	No
Respiratory abnormalities	No	No	No	No
Gastrointestinal abnormalities	No	No	Constipation	Constipation
Feeding difficulties	Yes	Yes	No	Yes
Organomegaly	No	No	No	No
Bowel and urinary incontinence	No	No	Yes	Yes

N/A, information not available; AEDs, anti‐epileptic drugs; MRI, magnetic resonance imaging; perc., percentile.

Patient 2 is a female patient born to consanguineous Lebanese parents. She had a severe developmental delay, brain malformation, irritability, orofacial dyskinesia, epilepsy, and failure to thrive at 2 years of age. The parents had a previous miscarriage and comparative genomic hybridization of the fetus revealed evidence of 45,XO chromosomes (Turner syndrome). The patient was born at full term (38 weeks) with a weight at 3325 g (65th percentile), length at 48 cm (15th percentile), and head circumference at 35 cm (71st percentile) without relevant abnormalities in perinatal adaptation. At the age of 6 months, she first showed epileptic spasms that were ultimately managed with triple treatment (vigabatrin, topiramate, and lamotrigine). EEG showed diffuse delta rhythm and hypsarrhythmia. While hypoplasia of corpus callosum was already detected in utero, brain MRI at 6 months of age showed dysgenesis of the corpus callosum with markedly hypoplastic and foreshortened body and underdeveloped rostrum, genu, and splenium, secondary radial configuration of the paramedian gyri with absent cingulate gyri, widened anterior interhemispheric fissure, hypoplastic anterior commissure and massa intermedia, agenesis of the septum pellucidum, asymmetrically rotated thalami, diffuse white matter volume loss, hypoplastic brainstem predominantly involving the pons, hypoplastic vermis with widening of the foramen of Magendie, hypoplastic superior and middle cerebellar peduncles, and a mega cisterna magna. The patient had constipation and failure to thrive with feeding difficulties, and no recurrent infections. Standard metabolic screenings showed no abnormalities.

### Molecular findings

A loss of function homozygous variant in *PI4K2A* NM_018425.4:c.925C>T, p.(Arg309Ter) was found in both patients from unrelated families, whereas the parents of each index patient showed heterozygous variants (Fig. 1A–C). This novel variant is not reported in multiple variant databases (gnomAD, UKbiobank, Centogene Bio/Databank, QSG, BRAVO TOPMed Freeze 8, GME Variome, Iranome) and leads to a protein truncation at amino acid residue 309 within the catalytic kinase domain.

### Functional studies of PI4K2A variants

**Figure 2:**
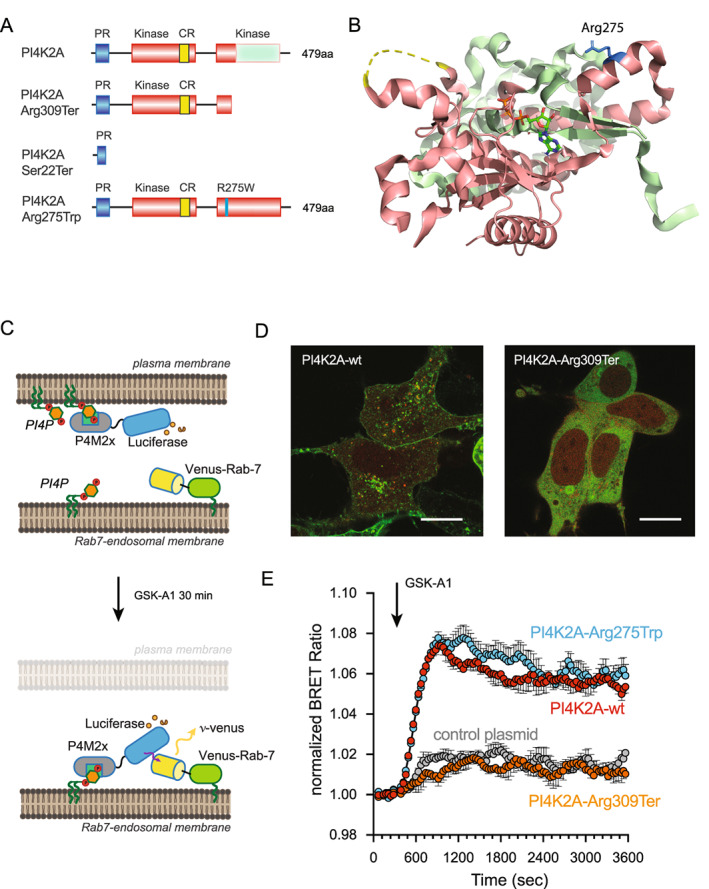
Characterization of PI4K2A variants using intact HEK293 cells. (A) Cartoon depiction of the PI4K2A sequence along with variants relevant to this study. The proline‐rich (PR; blue) and kinase (orange) domains, along with the palmitoylated cysteine‐rich CCPCC motif (CR; yellow), are specifically highlighted. (B) Ribbon representation of the solved PI4K2A structure in complex with adenosine trisphosphate^35^ (PDB Accession: 4PLA), with the membrane‐oriented surface facing upwards. Please note, as in (A), the truncated C‐Lobe of the kinase that is lost in the Arg309Ter variant has been colored green, and the side chain for residue Arg275 is highlighted as a stick representation in blue. (C) Schematic depicting the principle behind BRET‐based measurements of PI4P levels within Rab7‐positive endosomal compartments. Briefly, treatment of cells with the PI4KA‐selective inhibitor, GSK‐A1 (30 nmol/L), acutely reduces the PM levels of PI4P, which causes a well‐characterized PI4P‐sensitive reporter^17^ to drop from this compartment and find endosomal pools of PI4P that are made by PI4K isoforms, including PI4K2A, that are insensitive to GSK‐A1. For further information on this approach and important controls, please refer to previously published study.^17^ (D) Representative images comparing the subcellular localization of the wild‐type EGFP‐PI4K2A enzyme (left) with the EGFP‐PI4K2A‐Arg309Ter variants in HEK293‐AT1 cells (10 μm scale bars). (E) Kinetics of PI4P levels within the Rab7‐positive endosomal compartments of PI4K2A K/O cells expressing iRFP‐PI4K2A variants (wild‐type, truncated Arg309Ter), or the Arg275Trp substitution) or a vector control (iRFP‐C1) in response to treatment with GSK‐A1 (30 nmol/L). BRET measurements were made using the sLuc‐P4M2x‐T2A‐mVenus‐Rab7 biosensor^14^ and are presented as mean values ± SEM from three independent experiments carried out using triplicate wells. PI4P, phosphatidylinositol 4‐phosphate; PI4K, phosphatidylinositol 4‐kinase. [Colour figure can be viewed at wileyonlinelibrary.com]

To test the functionality of the mutated PI4K2A proteins, we generated GFP‐ and iRFP‐fused versions of the wild‐type and two homozygous variants (Arg275Trp and Arg309Ter) proteins. The Arg309Ter pathogenic variant was found in the patients described above, while the Arg275Trp variant was previously described in a patient with metabolic cutis laxa.^10^ Confocal microscopy showed that the GFP‐tagged truncated enzyme was fully cytoplasmic showing no association with any internal membranes, whereas the wild‐type protein was associated with the endosomal compartments as shown previously (Fig. 2D).^15^, ^16^ There was no obvious difference between the localizations of the wild‐type and the Arg275Trp substituted protein (not shown). In previous studies using PI4K2A K/O HEK293‐AT1 cells, we found that PI4K2A generates most of the PI4P in Rab7‐positive endo‐lysosomal compartments.^14^ In those studies, wildtype PI4K2A but not a catalytically inactive protein was able to restore PI4P levels in the Rab7‐positive endosomes. We used this system to test the functionality of the mutated forms of PI4K2A. The PI4P content of the Rab7 positive compartment was monitored by BRET that is based on the energy transfer between a Rab7‐targeted Venus and the Luciferase‐fused PI4P‐selecive protein binding module of Legionella (P4M‐2x) as described previously^14^, ^17^ and shown in the illustration (Fig. 2C). This approach also takes advantage of the use of GSK‐A1, a selective inhibitor of PI4KA, the enzyme that generates the PM pool of PI4P.^12^ Upon addition of GSK‐A1 to the cells, the PM pool of PI4P is slowly diminished causing the Luciferase‐fused PI4P binding module to be released from the PM and re‐localize to the other PI4P containing membranes including the Rab7 compartment as reflected in the increased BRET signal (Fig. 2E). As shown in Figure 2E, PI4K2A K/O cells show only a modest increase in the BRET signal monitored in the Rab7 compartment after GSK‐A1 addition (gray symbols) that has been attributed to the function of the other member of this enzyme family, PI4K2B.^14^ Expression of the wildtype iRFP‐fused PI4K2A substantially increased the amount of PI4P in the Rab7 compartment (red symbols). A similar increase was found with the Arg275Trp variant (blue symbols). In contrast, the truncated Arg309Ter variant of PI4K2A failed to restore the PI4P levels in the Rab7 compartment.

## Discussion

**Figure 3:**
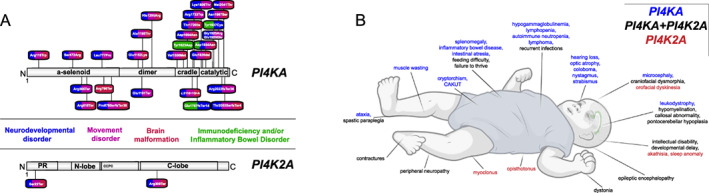
Summary of molecular and clinical findings in patients with Phosphatidylinositol 4‐Kinase Deficiency due to bi‐allelic variants in either *PI4KA* or *PI4K2A*. (A) *PI4KA* variants on protein level from three previously published studies.^6^, ^7^, ^8^ Bi‐allelic *PI4K2A* variants on protein level from one previously published study and our study.^9^ Missense/indel variants are found above protein illustrations, frameshift/stop variants below protein illustrations. Color coding relates to each symptoms/disorders in each patient that is associated to variant: bright blue neurodevelopmental disorder, pink – movement disorder, brown – brain malformation, green – immunodeficiency and/or inflammatory bowel disorder. Created with IBS Biocuckoo. (B) Comparison of phenotypes in PI4KA deficiency (blue), PI4K2A deficiency (red), and phenotypes found in both PI4K‐associated disorders (green) shows overlap specifically within the neurological disorders (dystonia, spastic paraplegia, contractures, peripheral neuropathy, epilepsy/epileptic encephalopathy, intellectual disability and/or global developmental delay, hypomyelination, callosal abnormalities, pontocerebellar hypoplasia) as well as craniofacial dysmorphia, failure to thrive, feeding difficulty, and recurrent infections. Created with BioRender.com. [Colour figure can be viewed at wileyonlinelibrary.com]

In this report we describe two cases with a bi‐allelic pathogenic variant resulting in the truncation of the PI4K2A protein. Taking into account the seminal paper reporting two patients from one family,^9^ our findings establish that PI4K2A deficiency is a novel cause of developmental encephalopathy with epilepsy and hyperkinetic movement disorders^18^ (Fig. 3A). Our results from Figure 2 clearly showed that the truncated PI4K2A protein was completely inactive, which is not surprising given the fact that half of its catalytic domain was missing (Fig. 2B). It is also likely that such truncated protein is unstable and would be degraded in the patients' cells. The genome aggregation database (gnomAD) lists the gene *PI4K2A* with a very high probability of being intolerant towards loss‐of‐function (pLoF pLI = 0.8) which strengthens the case that null variants are not tolerated in the healthy population. In contrast, we found that the Arg275Trp substituted enzyme was fully functional in our experimental conditions testing the protein *in situ* within cells. This variant was previously proposed as a possible disease‐causing allele in a patient with metabolic cutis laxa although the patient also carried a homozygous missense variant in the Twinkle mtDNA helicase gene (*TWNK*).^10^ The patient reported in that study also showed lipodystrophy, microcephaly, muscular hypotonia, global developmental delay, choreoathetosis, brain malformations, progeroid facial appearance, and hepatomegaly. All of these symptoms have been documented in previous reports of TWNK deficiency.^10^ Importantly, neither of our patients with PI4K2A deficiency showed any signs of cutis laxa or joint hypermobility. The findings from our cellular experiments raise the possibility that the missense variant in *PI4K2A* with a substitution in Arg275Trp described in that study may not have been the primary cause of the disease, although we cannot rule out that the PI4K2A Arg275Trp variant has subtle functional alterations not revealed by our assays, which also contributed to the clinical manifestation found in that patient.

It is notable that significant overlap exists in pathogenic variants in *PI4KA* and *PI4K2A* with phenotypes including severe combined NDD, epilepsy or even epileptic encephalopathy, brain malformation (hypomyelination/atrophy, corpus callosum abnormalities, pontocerebellar hypoplasia^8^), movement disorders (dystonia, spastic paraplegia), craniofacial dysmorphia, and feeding difficulties with failure to thrive (Fig. 3B). Patients with variants in *PI4KA* or *PI4K2A* share common features of severe developmental encephalopathy reflecting diffuse neurological dysfunction.^6^, ^7^, ^8^, ^9^ Brain malformation, severe psychomotor retardation, epilepsy, movement disorders, contractures, spasticity and peripheral nervous system involvement have been described in both defects^6^, ^7^, ^8^, ^9^ (Fig. 3B). Movement disorders and neuropathy have been well established in patients with monogenic deficiencies in intracellular trafficking.^19^ Severe neurological phenotypes with optic atrophy, epileptic encephalopathy or coloboma also presented with PID or IBD in several patients with *PI4KA* variants (Fig. 3A).^6^ It is unclear to what extent immunodeficiency triggers a severe deterioration of the neurological phenotype.

Immunological features in patients with *PI4KA* variants include T‐cell lymphopenia primarily affecting CD8+ T cells, B‐ and NK cell lymphopenia, a−/hypogammaglobulinemia, splenomegaly, and poor antibody responses to previous immunizations.^6^ A 10‐year‐old girl with PI4KA deficiency had an only moderate lymphopenia with marked reduction in B and NK cells without history of recurrent infections. Of note, she showed a diffuse grade 3A follicular non‐Hodgkin lymphoma with a bcl6 translocation. One of our *PI4K2A* cases had recurrent infections with multiple hospitalizations. However, the patient died before diagnostic work‐up for a suspected immunodeficiency. As PID is a recurring feature in the cohorts of published cases with *PI4KA* variants (*n* = 8/22), PID may be underreported in cases with *PI4K2A* variants due to the much lower number of cases. Further studies are required to lay the groundwork for genotype–phenotype correlations in patients with pathogenic variants in *PI4K2A*.^20^, ^21^, ^22^

We observed major clinical overlaps with other disorders within the emerging group of innate errors in intracellular trafficking that is likely explained by the prominent roles of PI4KA and PI4K2A in the control of intracellular trafficking pathways. A recent study revealed an important role of PI4K2A in Rab7‐positive compartments supporting the fusion of lysosomes with autophagosomes.^14^ Another recent study reported bi‐allelic variants in the vacuolar protein sorting‐associated protein 41 homolog gene (*VPS41*) in patients suffering from progressive neurodevelopmental disease comprising cognitive impairment, cerebellar atrophy/hypoplasia, motor dysfunction with ataxia and dystonia, and nystagmus due to lysosomal deregulation.^23^ VPS41 is part of the homotypic fusion and vacuole protein sorting (HOPS) complex which tethers the complex to Rab7‐positive endosomes and support lysosomal function. Heterozygous variants in the vacuolar protein sorting‐associated protein 16 homolog gene (*VPS16*), another member of the HOPS complex, were also linked to early‐onset progressive dystonia with predominant cervical, bulbar, orofacial, and upper limb involvement.^24^ Patients with bi‐allelic variants in *VPS16* showed a more severe phenotype with similar features to PI4K deficiency, including delayed myelination, brain atrophy, neutropenia, skeletal abnormalities, and dysmorphic features.^25^ Defects in Rab7‐mediated vesicular trafficking were also found in other neurodegenerative movement disorders, such as in models of Parkinson's disease for the clearance of toxic protein aggregate alpha‐synuclein.^26^, ^27^ Another example of dysfunctional Rab7‐mediated signaling in NDD is found in patients with Vici syndrome and recessive variants in the ectopic P‐granules autophagy protein 5 (*EPG5*) gene,^28^ where EPG5 deficiency causes a blockade in autophagosome‐lysosome tethering and deficient Rab7‐mediated signaling.^29^, ^30^ Patients with Vici syndrome also show microcephaly, corpus callosum abnormalities, and various movement disorders such as spastic paraplegia, similarly to our patients with PI4K2A deficiency. This spectrum of movement disorders was also observed in patients with PI4KA deficiency, including dystonia, spastic paraplegia, opisthotonus, ataxia, and myoclonus. The patients described here with pathogenic variants in *PI4K2A* show spastic paraplegia, orofacial dyskinesia, and intermittent dystonic postures of the upper limbs with clenched fist.

PI4K2A is known to form a tripartite complex with the biogenesis of lysosome‐related organelles complex 1 (BLOC‐1) and adaptor‐related protein complex 3 (AP‐3) that is crucial for the endocytic pathway.^31^ Pathogenic variants in both *BLOC1* or AP‐3 complex (*AP3B1* and *AP3D1*) are associated with Hermansky‐Pudlak syndrome, in which neurodevelopmental and non‐neuronal phenotypes (albinism, PID) result from lysosomal defects. Sorting motif and kinase‐active sites in PI4K2A were found to be required for its localization to LAMP‐1–positive endosomes^32^ and for the biogenesis of lysosomes by directing the sorting of membrane proteins to lysosomes.^33^ One patient with a *PI4KA* variant showed widespread small‐pigmented skin macules,^6^ however there is no report of patients with a *PI4K2A* variant and PID or pigmentation abnormalities.

As outlined above, there is a remarkable overlap in the phenotypes of patients with pathogenic variants in *PI4KA* and *PI4K2A*, namely brain malformations, epilepsy and movement disorders. Further clinical studies are required to lay the groundwork for natural history of the disease, genotype–phenotype correlations, and specific diagnostics to allow for timely diagnosis, informed management and anticipatory guidance in an underreported and ultra‐rare disorder.^34^

## Conflict of Interests

S. K. and A. M. B.‐A. are employes of CENTOGENE GmbH. All other authors have nothing to declare.

## Supporting information


**Video S1.** Patient 1 from family showing intermittent orofacial dyskinesia and dystonic postures of the upper limbs with clenched fist and thumb adduction.
